# Supplementary material for: Navigability of temporal networks in hyperbolic space
Source: Sci Rep. 2017 Nov 8;7:15054. doi: 10.1038/s41598-017-15041-0 (PMC5678097; doi:10.1038/s41598-017-15041-0)
Supplement: Supplementary file 1 — Supplementary Information [file 41598_2017_15041_MOESM1_ESM.pdf]

Supplementary information for: Navigability of temporal networks in hyperbolic space

Elisenda Ortiz,[1,2] Michele Starnini,[1,2] and M. Ángeles Serrano[1,2,3,*]

[1]*Departament de Física de la Matèria Condensada, Universitat de Barcelona, Martí i Franquès 1, 08028 Barcelona, Spain*

[2]*Universitat de Barcelona Institute of Complex Systems (UBICS), Universitat de Barcelona, Barcelona, Spain*

[3]*ICREA, Pg. Lluís Companys 23, E-08010 Barcelona, Spain*

(Dated: October 13, 2017)

I. GREEDY ROUTING IN HYPERBOLIC TEMPORAL MAPS

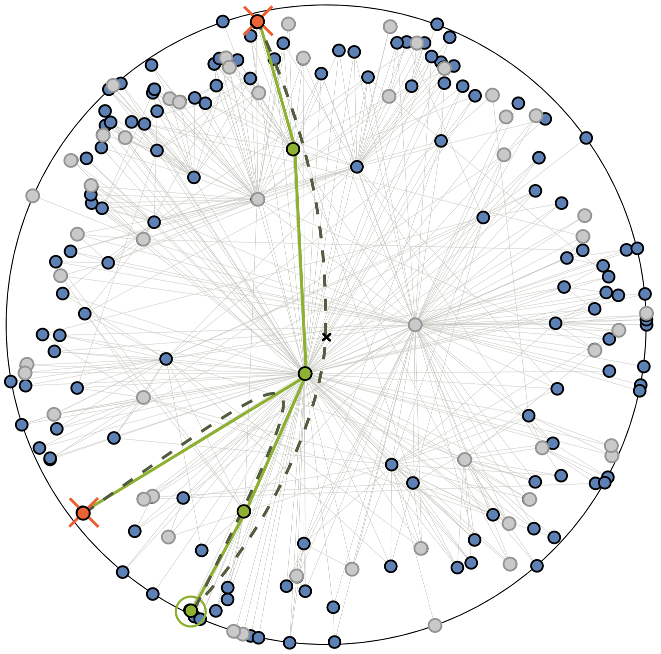

Figure S1. **Visualization of the WTW temporal map $\mathcal{M}(\mathcal{G}, S)$ in the hyperbolic plane and greedy routig in it.** Inactive nodes at time t appear in grey while blue color is reserved for active ones. The two dashed lines correspond to two geodesics between the source node, circled in green at the bottom, and two destinations represented by red nodes with crosses. The solid green lines, which are the greedy paths between the same source-destination nodes, are remarkably congruent with the geodesics. In this example, if a green node along a greedy path (specially the most centric one) is switched off at the time of receiving a packet, it will force the greedy path to deviate from the geodesic and turn it into a longer greedy path.

* marian.serrano@ub.edu

II. ACTIVATION OF NODES WITHIN DEGREE INTERVALS

In this section the random activation-inactivation dynamics is targeted to subsets of equal number of nodes whose degrees belong to a certain degree interval. Figure. S2 shows the p_s and $\overline{s}$, measured when only one of these subsets of nodes is activated with constant $a < 1$ and the rest of the network remains active ($a = 1$). The dynamic subsets are identified by their average degree $\overline{k}$ and are of size $\xi = 5\%$ of the total number of nodes N. Results from Fig. S2 imply that switching on and off nodes with low degree has a limited effect on the navigability efficiency. Moreover, when the dynamic subset is composed by nodes of any degree, the obtained p_s remains low and close to the static (see black diamonds, Fig. S2) demonstrating that the incresed success achived by temporal networks relies on the activity of densely connected nodes.

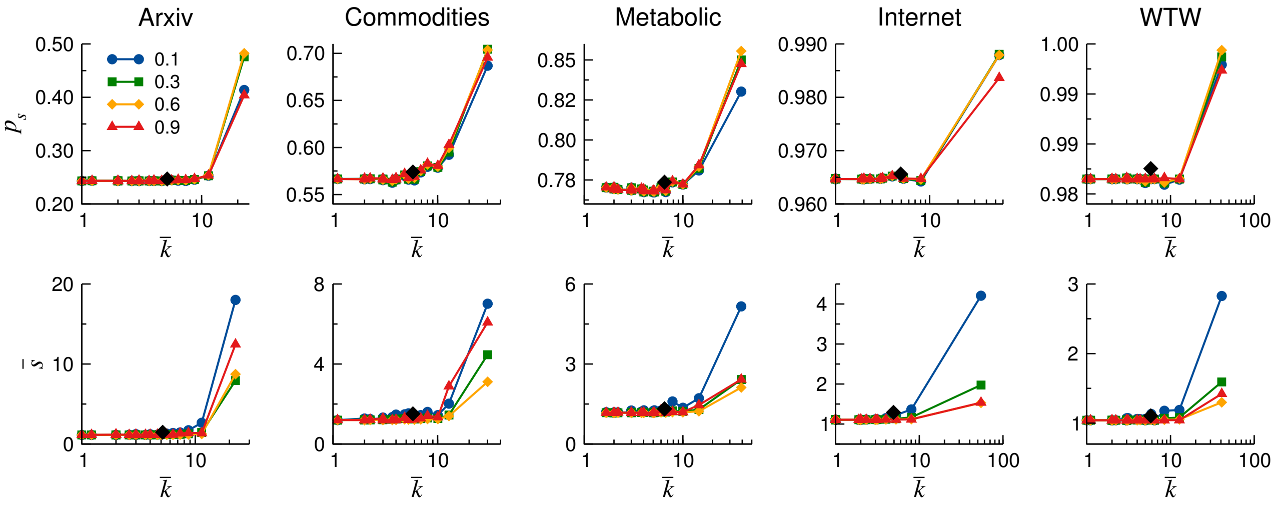

Figure S2. **Success ratio p_s (top row) and average stretch $\overline{s}$ (bottom row) as a function of the average degree $\overline{k}$ of node bins** with temporal behaviour, for different values of the activation probability a. The success ratio is measured in a fully active network, where only nodes from a single bin of size $\xi = 5\%$ can activate with probability $a < 1$. The black diamonds correspond to the case of selecting the number of nodes that activate with $a < 1$ uniformly at random from the whole network and averaging over 10^3 realizations. In this later case, the results for different a's overlap, so for clarity the black diamonds are displayed only for $a = 0.1$.

III. LINEAR ACTIVATION DYNAMICS OF NODES

In this section, we set the activation probabilities to be linearly dependent upon the degree (k) of the nodes. This is, $a(k) \in [0, 1]$ takes the form:

$$a(k) = bk + c \tag{1}$$

We tune the average activation probability $\overline{a} = \frac{1}{N}\sum_{i=1}^{N} a(k)_i$ of the whole network, so for each $\overline{a}$ we calculate the corresponding coefficients b and c taking into account that $\overline{a}$ is set constant. Then, we activate each node proportionally to its degree following (1). The coefficients b and c are obtained as follows.

Given a fixed $\overline{a}$ value, we have that

$$\overline{a} = \frac{1}{N}\sum_{i=1}^{N}(bk_i + c)$$

$$c = \overline{a} - \frac{b}{N}\sum_{i=1}^{N} k_i$$

$$c = \overline{a} - b\overline{k} \tag{2}$$

The maximum ($a_{\max}$) and minimum ($a_{\min}$) values of $a(k)$ are related with $k_{\max}$ and $k_{\min}$ through the equation of a line, see Fig. S3a. Moreover, recall that $k_{\min}$, $k_{\max}$ are fixed numbers since they are attributes of the network, and $a_{\min}$, $a_{\max} \in [0, 1]$ because $a(k)$ is a probability. Therefore, it is satisfied that

- $\underline{b > 0}$: $$\begin{aligned} a_{\max} &= bk_{\max} + c \\ &= bk_{\max} + \overline{a} - b\overline{k} \\ &= \overline{a} + b(k_{\max} - \overline{k}) \end{aligned} \qquad \begin{aligned} a_{\min} &= bk_{\min} + c \\ &= \overline{a} + b(k_{\min} - \overline{k}) \end{aligned}$$

- $\underline{b < 0}$: $$a_{\max} = \overline{a} - |b|(k_{\min} - \overline{k}) \qquad a_{\min} = \overline{a} - |b|(k_{\max} - \overline{k})$$

If we now apply $a_{\max} \leq 1$ and $a_{\min} \geq 0$ in order to ensure that $a(k) \in [0, 1]$,

- $\underline{b > 0}$: $$\overline{a} + b(k_{\max} - \overline{k}) \leq 1 \qquad \overline{a} + b(k_{\min} - \overline{k}) \geq 0$$ $$b \leq \frac{1 - \overline{a}}{(k_{\max} - \overline{k})} \qquad b \leq -\frac{\overline{a}}{(k_{\min} - \overline{k})}$$

- $\underline{b < 0}$: $$\overline{a} - |b|(k_{\min} - \overline{k}) \leq 1 \qquad \overline{a} - |b|(k_{\max} - \overline{k}) \geq 0$$ $$|b| \leq -\frac{(1 - \overline{a})}{(k_{\min} - \overline{k})} \qquad |b| \leq \frac{\overline{a}}{(k_{\max} - \overline{k})}$$

From the above conditions we obtain that b must satisfy

$$b \leq \min\left\{ \frac{1 - \overline{a}}{(k_{\max} - \overline{k})}, \frac{-\overline{a}}{(k_{\min} - \overline{k})} \right\} \qquad \text{if} \quad b > 0 \tag{3}$$
$$|b| \leq \min\left\{ \frac{-(1 - \overline{a})}{(k_{\min} - \overline{k})}, \frac{\overline{a}}{(k_{\max} - \overline{k})} \right\} \qquad \text{if} \quad b < 0. \tag{4}$$

Therefore, we can select any b values that satisfy the above inequalities. Afterwards, the coefficient c is readily found from Eq. 2. We take one positive and one negative b, so to maximize the heterogeneity of the activation dynamics, as shown in Fig. S3b. By choosing b that correspond to the equality sign in (3) and (4) we fully activate ($b > 0$) or inactivate ($b < 0$) the main hub. For this particular case, one can observe the resulting p_s and $\overline{s}$ in Fig. S5. Since we noticed that strongly hierarchical networks such as the Internet and the WTW present a singular behaviour when the main hub is removed from the routing ($b < 0$), we exclude this choice of coefficient and set the minimum activation probability for the main hub to be 10^{-3}, and simetrically the maximum to $1 - 10^{-3}$. Figure S4 shows the results of linear activation of nodes for a an average activation probability of $\overline{a} = 0.3$, different from the one employed in the main text ($\overline{a} = 0.5$). In this case, all temporal maps exhibit the same trends as before, both for p_s and $\overline{s}$, implying that qualitatively, the effects induced by linear activation are independent of $\overline{a}$.

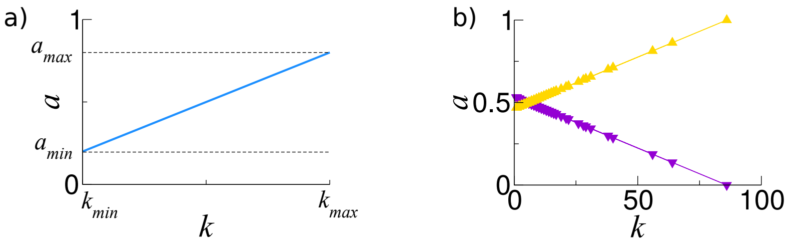

Figure S3. **a)** In blue, example of a linear activation probability. **b)** Example, from Commodities network, of the two curves $a(k)$, used for linearly activating the nodes when $\overline{a} = 0.5$. In yellow $a(k)$ with $b > 0$ and in purple with $b < 0$. The minimum activation probability is explicitly set to not to be exactly 0 but of order 10^{-3}; simetrically the maximum activation corresponds to $a \approx 1 - 10^{-3}$.

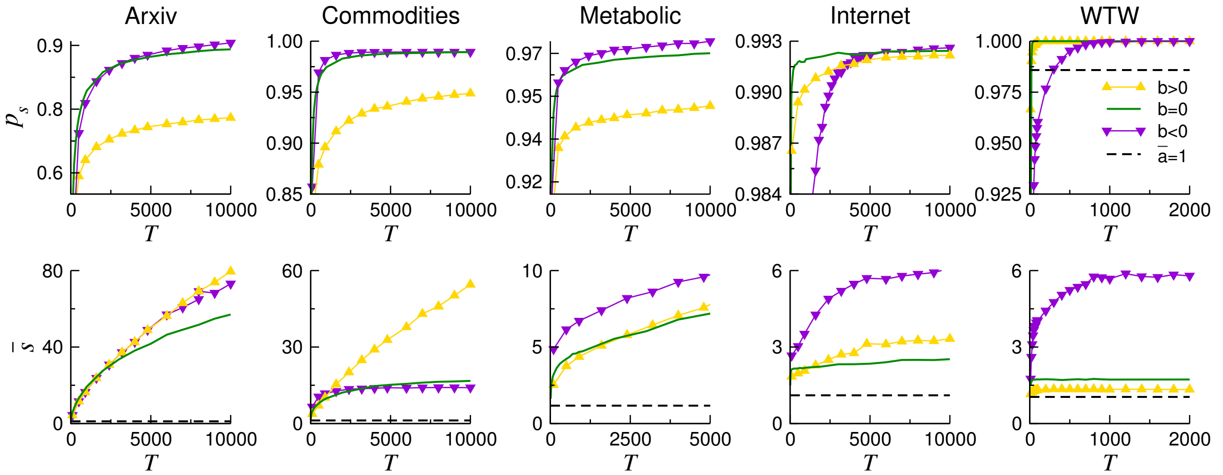

Figure S4. **Success ratio** p_s **(top row) and average stretch** $\bar{s}$ **(bottom row) as a function of** T, for $\bar{a} = 0.3$, in five temporal maps $\mathcal{M}(\mathcal{G}, S)$. Solid lines designate constant activation of nodes, while symbols indicate nodes linearly activate following (1); proportionally to their k ($b > 0$), or inversely proportional ($b < 0$). In dashed line, p_s and $\bar{s}$ corresponding to greedy routing in the static maps $\mathcal{M}(G_0, S)$.

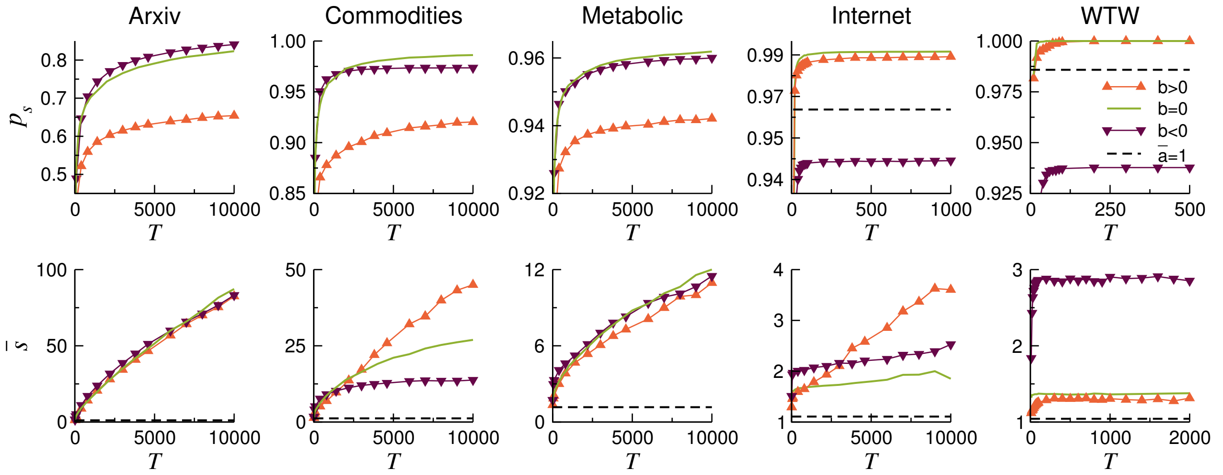

Figure S5. **Success ratio** p_s **(top row) and average stretch** $\bar{s}$ **(bottom row) as a function of** T, for $\bar{a} = 0.5$, in five temporal maps $\mathcal{M}(\mathcal{G}, S)$. In this case, the minimum activation of main hub is allowed to be null and the maximum exactly 1. Solid lines designate constant activation of nodes, while symbols indicate nodes linearly activate following (1); proportionally to their k ($b > 0$), or inversely proportional ($b < 0$). In dashed line, p_s and $\bar{s}$ corresponding to greedy routing in static maps $\mathcal{M}(G_0, S)$. Notice the low p_s and $\bar{s}$ achieved by the Internet and the WTW when $b < 0$, due to the main hub being always totally inactive during the routing process.

IV. AVERAGE GEOMETRIC STRETCH

The average geometric stretch $\bar{s}_g$ of successful greedy paths, is defined as the ratio between the cummulative hyperbolic distance from the hop-length of greedy paths and the corresponding geodesics. The $\bar{s}_g$ tells us how much the successful greedy paths elongate with respect to the shortest ones in the metric space.

	ArXiv	Commodities	Metabolic	Internet	WTW
$\bar{s}_g$	3.10	2.33	2.38	2.39	1.35

Table S1. **Average geometric stretch of five static maps** $\mathcal{M}(G_0, S)$**.** The values were obtained averaging over a number of random source–destination pairs, that is the minimum between 10^5 and $N(N-1)/2$, where N is the number of nodes of the network.

An inescapable consequence of embedding the network topologies in the hyperbolic space is the fact that very often there exist nodes which share the precise same coordinates. These nodes that occupy the exact same position in the hyperbolic disk were not considered as possible source–destination pairs, since the geodesic connecting them is of length 0, thus making $\bar{s}_g$ to diverge. The average geometric stretch for temporal maps $\mathcal{M}(\mathcal{G}, S)$ was computed taking only successful paths from a representative subset of randomly chosen node pairs. For each network the number of source–destination pairs was set to be the lowest number between $N(N-1)/2$ and 10^5 , and was changed at each different T. The behaviour observed for $\bar{s}_g$ is qualitatively similar to the observed for the topological average stretch in Fig.1, in the main paper. This means, *i)* the geometric lengths of greedy paths elongate more for temporal networks with lower static success, due to the breaking of a greater number of topological traps *ii)* the closest the activation probability is to the optimal, a_O, the less the greedy paths elongate with respect to geodesics.

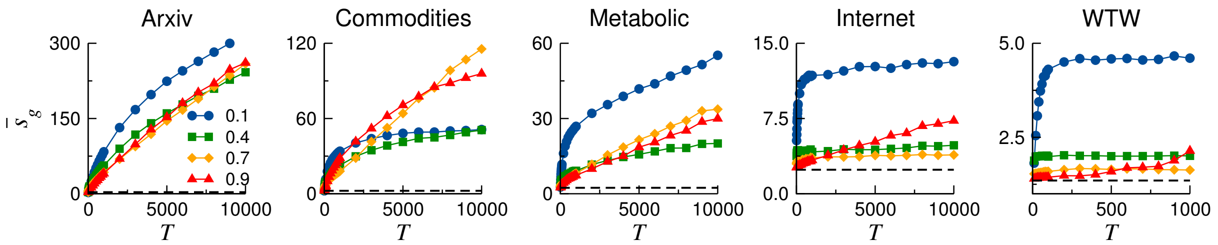

Figure S6. **Average geometric stretch $\bar{s}$ as a function of** T, for different values of the activation probability a, in five temporal maps $\mathcal{M}(\mathcal{G}, S)$. Average geometric stretch for $a = 1$, corresponding to greedy routing in the static maps, is plotted in dashed line.

V. COVERAGE

The average coverage $\bar{\kappa}$, tells the average number of *different* visited nodes, against the average number of nodes that constitute a successful path. This metric measures how much the information packet travels around the network (and the metric space). Figure S7 shows that for low activation probability ($a = 0.1$), the information packet jumps among a great number of different nodes, whereas for high activation ($a = 0.9$) it only visits a few. By lowering the activity of the nodes, the information packet is forced to jump often to any available position, hence we find that the longer the path the higher the coverage. On the contrary, when most nodes remain almost invariably active, the longer path lengths displaying low $\bar{\kappa}$ confirm the idea that the packet is found moving in cycles, failing to scape a topological trap. For the static references, plotted as solid lines in Fig. S7, we observe straight lines of slope 1. In the static case, a path is always declared unsuccessful if a node is tried to be visited twice. Therefore, the average path length always coincides with the number of different visited nodes, thus producing unitary slopes. Nonetheless, the solid lines inform us about the maximum average path length that one can expect from a network only due to its own structure.

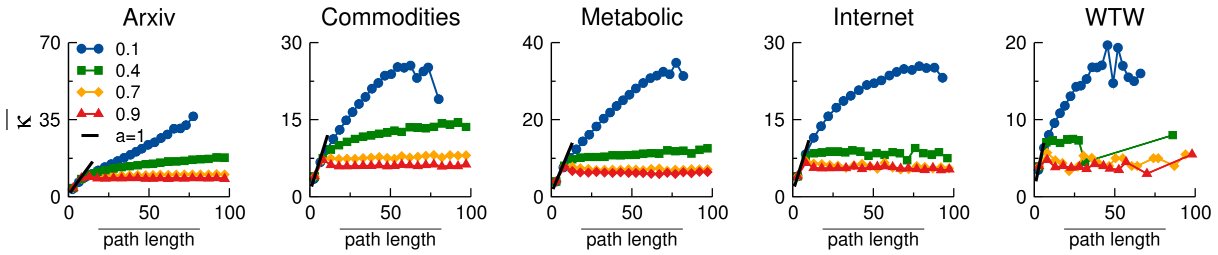

Figure S7. **Average coverage $\bar{\kappa}$ as a function of the average path length of successful paths** for different values of the activation probability in five temporal maps $\mathcal{M}(\mathcal{G}, S)$. The black solid lines correspond to $a = 1$, which indicates the greedy routing is performed on static maps.
